# Supplementary figures and images for: Regeneration of starfish radial nerve cord restores animal mobility and unveils a new coelomocyte population
Source: Cell Tissue Res. 2023 Aug 22;394(2):293–308. doi: 10.1007/s00441-023-03818-x (PMC10638123; doi:10.1007/s00441-023-03818-x)

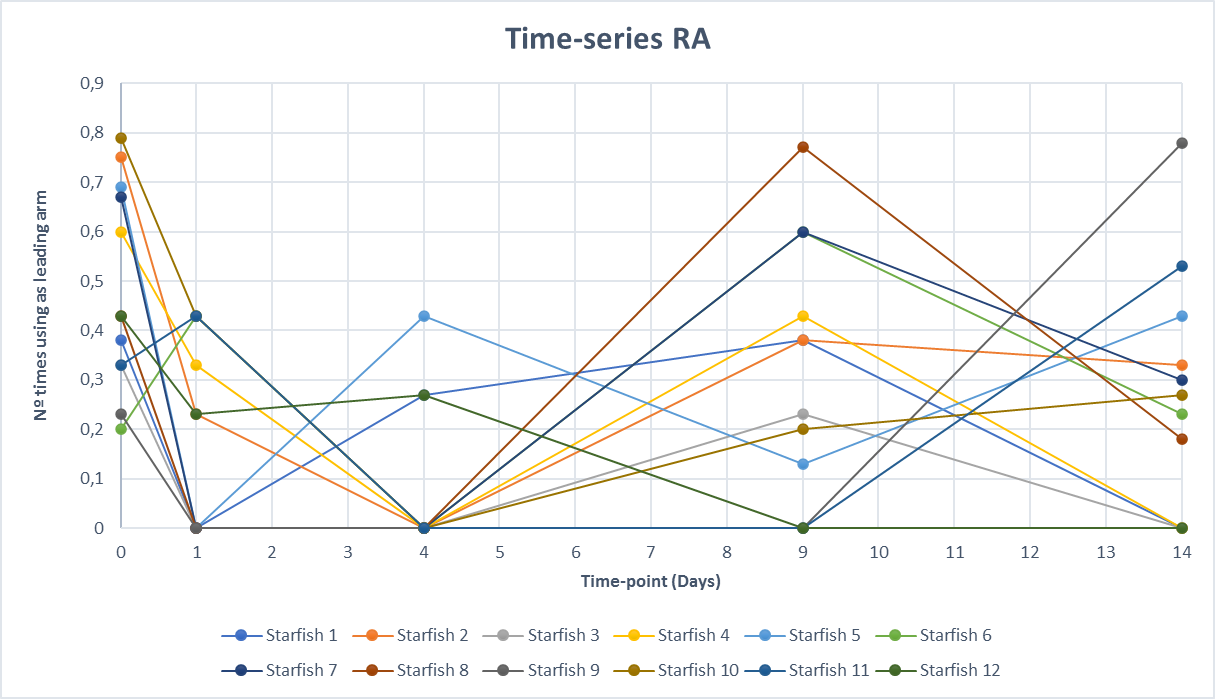


Supplementary Figure 1. Time series plot of the RN usage as leading arm by each starfish.

Supplement: Supplementary file 1 — Online Resource 1 Supplementary Fig. 1. Time series plot of the RN usage as leading arm by each starfish (DOCX 127 KB) [file 441_2023_3818_MOESM1_ESM.docx]
