# Supplementary material for: Regeneration of starfish radial nerve cord restores animal mobility and unveils a new coelomocyte population
Source: Cell Tissue Res. 2023 Aug 22;394(2):293–308. doi: 10.1007/s00441-023-03818-x (PMC10638123; doi:10.1007/s00441-023-03818-x)
Supplement: Supplementary file 2 — Online Resource 2 Supplementary information: Gating strategy for flow cytometry (DOCX 91 KB) [file 441_2023_3818_MOESM2_ESM.docx]

**Supplementary information**

Gating strategy of FC results firstly passed by the selection of all the positive events in the DRAQ5 channel that led to the exclusion of dead cells and high auto-fluorescent cells. Then, the relation between SSC-H (height) and FSC-A (area), and between SSC-A (area) and SSC-H was used to select the single cells and remove the aggregates (Supplementary figure 2).


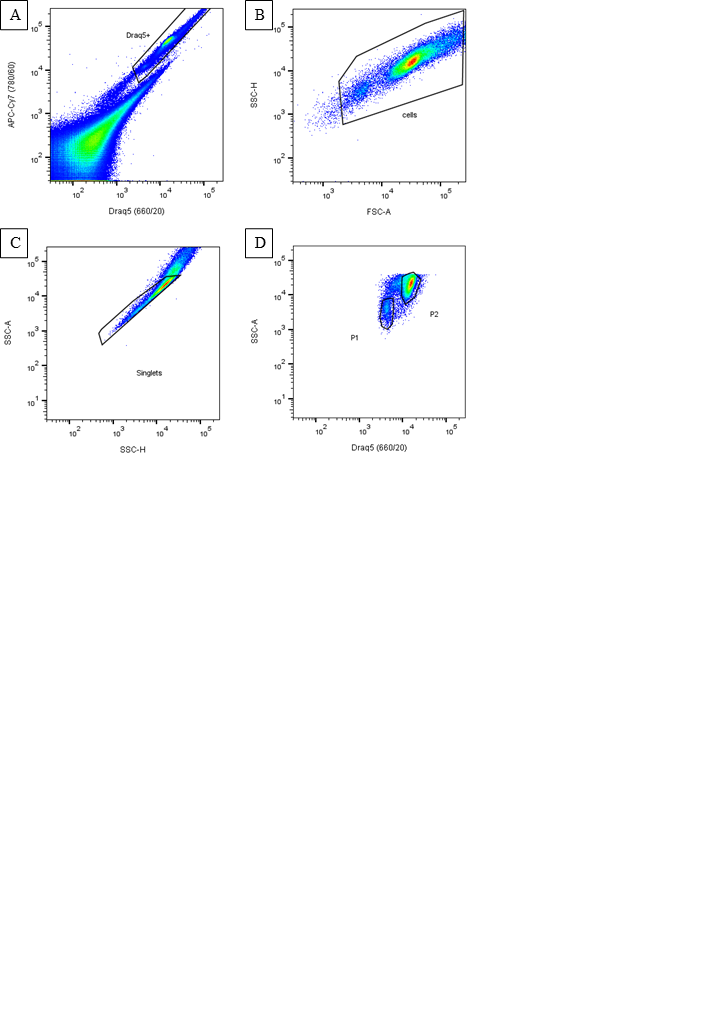


**Supplementary figure 2.** Representative staining and gating strategy of totally circulating coelomocytes. Cell population was selected by successive gating: (A) exclusion of high auto-fluorescent cells through the selection of DRAQ5 labeled cells; (B) exclusion of debris and some dead cells through the relation between SSC-H (height) and FSC-A (area); (C) removal of aggregates and selection of single cells through the relation between SSC-A and SSC-H and (D) P1,P2 and P3 populations selection by looking at median fluorescence intensity in DRAQ5 channel vs SSC-A.
